# Special Issue “Molecular Insights into Reproductive Biology and Related Diseases”

**DOI:** 10.3390/ijms27136003

**Published:** 2026-07-03

**Authors:** Alejandro Vicente-Carrillo

**Affiliations:** Department of Animal Production, Veterinary Faculty, Complutense University of Madrid, 28040 Madrid, Spain; alevic05@ucm.es

Reproductive biology relies on a complex and highly coordinated network of molecular and cellular processes that regulate gametogenesis, fertilization, and early embryonic development. Disruptions in these finely tuned mechanisms may lead to infertility and a wide range of reproductive disorders affecting both humans and animals. In recent years, advances in molecular biology and omics technologies have significantly improved our understanding of these processes, providing new insights into the mechanisms underlying reproductive function and dysfunction.

The Special Issue entitled “Molecular Insights into Reproductive Biology and Related Diseases” brings together a collection of original research articles and reviews that address key aspects of reproductive biology from a molecular perspective. The contributions included in this issue reflect the diversity of the field, encompassing studies on spermatogenesis, sperm function, oocyte competence, embryo development, and reproductive pathologies across different species.

Several articles in this Special Issue focus on the molecular regulation of male reproduction. In this context, Buev et al. [contribution 1] investigated the role of NSUN7, a testis-specific RNA methyltransferase, in spermiogenesis. Through proteomic profiling, the authors demonstrated that NSUN7 is essential for proper sperm flagella formation, with its disruption leading to structural defects in the fibrous sheath, impaired sperm motility, and male infertility. These findings provide important insights into the molecular mechanisms governing sperm structural integrity and function.

Complementing these findings, Jiang et al. [contribution 2] reviewed the complex regulatory networks of pre-mRNA alternative splicing during mammalian spermatogenesis. Their work highlights the critical role of alternative splicing in ensuring the precise temporal and spatial expression of genes required for germ cell development, emphasizing its importance as a key regulatory mechanism in male fertility.

Additional studies further explore sperm function and pathology in animal models. Rafalska et al. [contribution 3] examined the phosphoproteomic profile of turkey spermatozoa in relation to semen type, particularly in the context of yellow semen syndrome. Their findings reveal significant alterations in protein phosphorylation patterns associated with reduced semen quality and fertility, providing new molecular insights into this emerging reproductive disorder. Similarly, Rehder et al. [contribution 4] investigated the role of Sertoli cell dysfunction in dogs with chronic asymptomatic orchitis. Their results demonstrate that altered Sertoli cell function contributes to spermatogenic arrest, immune cell infiltration, and disruption of the blood–testis barrier, thereby highlighting the central role of the testicular microenvironment in maintaining spermatogenesis.

Female reproductive biology and fertility are also addressed in this Special Issue. Didžiokaitė et al. [contribution 5] evaluated the potential of serum vascular endothelial growth factor (VEGF) as a biomarker for unexplained infertility in women. Their study suggests that VEGF may serve as a promising diagnostic marker, while also indicating that immune-related conditions such as atopy may influence its levels, thus adding complexity to its clinical application.

In addition to human studies, applied reproductive strategies in animal production are explored. Vargas Ortiz et al. [contribution 6] assessed the effects of two hormonal protocols for fixed-time artificial insemination in repeat breeder dairy cows. Their findings provide valuable information on follicular dynamics, hormonal responses, and fertility outcomes, contributing to the optimization of reproductive management in livestock.

The Special Issue also includes a comprehensive review by Gałęska et al. [contribution 7] focusing on the role of mitochondrial processes in oocyte maturation and embryo culture. This review underscores the importance of mitochondrial function in determining oocyte quality, developmental competence, and embryo viability, highlighting mitochondria as central regulators of reproductive success and potential targets for improving assisted reproductive technologies.

Another important contribution to this Special Issue addresses the molecular mechanisms underlying sperm function and male fertility regulation. Torres Juárez et al. [contribution 8] investigated the effects of pimozide, an FDA-approved drug, on human sperm physiology, focusing on its interaction with the CatSper calcium channel. The authors demonstrated that pimozide inhibits CatSper-mediated calcium influx, leading to impaired sperm hyperactivation and a reduced acrosome reaction, both of which are essential processes for successful fertilization. These findings provide valuable insights into the molecular regulation of sperm function and identify CatSper as a potential target for the development of novel non-hormonal male contraceptive strategies.

Taken together, the contributions included in this Special Issue provide a comprehensive overview of current advances in reproductive biology at the molecular level. They highlight the importance of integrating basic research with clinical and applied approaches, as well as the value of comparative studies across species. The insights gained from these works not only deepen our understanding of reproductive mechanisms but also pave the way for the development of novel diagnostic tools and therapeutic strategies for reproductive disorders. Hopefully, collection will serve as a useful resource for researchers and clinicians and will stimulate further advances in the field of reproductive biology.

